# Fatty Acid and Micronutrient Profile of Longissimus Lumborum from Red Angus and Red Angus x Akaushi Cattle Finished on Grass or Grain

**DOI:** 10.3390/foods11213451

**Published:** 2022-10-31

**Authors:** Lucas Krusinski, Isabella C. F. Maciel, Selin Sergin, Travis Goeden, Jeannine P. Schweihofer, Sukhdeep Singh, Jason E. Rowntree, Jenifer I. Fenton

**Affiliations:** 1 Department of Food Science and Human Nutrition, Michigan State University, 469 Wilson Rd, East Lansing, MI 48824, USA; 2 Department of Animal Science, Michigan State University, 474 S Shaw Ln, East Lansing, MI 48824, USA; 3 Michigan State University Extension, 200 Grand River Ave, Port Huron, MI 48060, USA; 4 Department of Plant, Soil, and Microbial Sciences, Michigan State University, 1066 Bogue St., East Lansing, MI 48824, USA

**Keywords:** pasture, grass-finished beef, omega-3 fatty acids, vitamin E, diet, minerals

## Abstract

Cattle diet and breed modify the nutritional profile of beef. The objective of this study was to compare the fatty acid (FA) and micronutrient profiles of Red Angus (RA) and RA x Akaushi (AK) crossbreed steers fed either a grass or grain diet. This two-year study randomly assigned steers to the diets using a 2 × 2 factorial experiment. FAs and micronutrients were analyzed. Diet effect was the strongest with grass-finished beef being higher in *n*-3 polyunsaturated FAs (*p* < 0.001), conjugated linoleic acid (*p* < 0.05), vaccenic acid (*p* < 0.05), iron (*p* < 0.001), and vitamin E (*p* < 0.001) compared to grain-finished beef. Breed effects were observed for lauric and myristic acids (*p* < 0.05), selenium (*p* < 0.05), and zinc (*p* < 0.01) with AK containing more of these compounds than RA. Diet × breed effects were non-existent. These results indicate that diet has a stronger influence than breed on modifying the nutritional profile of beef. Because of a more favorable FA and antioxidant profile, consumption of grass-finished beef could benefit human health.

## 1. Introduction

Meat is an important part of the diet of many cultures and is consumed for its taste and nutritional properties [1,2]. Beef consumption increases with population and per capita income [3]. World meat production was estimated at 328 Mt in 2020 and is expected to reach 374 Mt in 2030 [4]. Beef is highly nutrient-dense and provides significant amounts of protein, fat, zinc, iron, selenium, and B vitamins [5]. Beef is also an important source of conjugated linoleic acid (CLA), and depending on the production system, can provide beneficial omega-3 (*n*-3) polyunsaturated fatty acids (PUFAs) mainly as alpha-linolenic acid (ALA) which is a precursor for eicosapentaenoic acid (EPA), docosapentaenoic acid (DPA), and docosahexaenoic acid (DHA) [6]. Cattle diet and breed are two factors that modify the nutritional profile of beef [1]. Feeding cattle a diet of either grass or grain or selecting for different breeds can alter the fatty acid (FA) and micronutrient content of beef [6,7].

Cattle diet significantly impacts the nutritional profile of beef, leading to variations in the FA profile [8,9,10]. The nutrient profile of grass-finished beef (GFB), compared to grain-finished beef, is generally more consistent with health recommendations, especially regarding *n*-3 PUFAs and phytochemicals [5,11,12]. GFB is usually higher in CLA, *n*-3 PUFAs, vitamin E and beta-carotene, and has a more favorable *n*-6:*n*-3 ratio compared to conventional beef [1,13,14,15]. On the other hand, grain-finished beef contains more *cis*-monounsaturated FAs (MUFAs), especially oleic acid (C18:1 *c*9) [16,17]. The nutritional profile of GFB may vary with different plant species, supplemental feeds, regions, and season of slaughter [15,18,19]. 

Crossbreeding is a common practice used to produce calves for fattening and finishing while benefiting from hybrid vigor [20]. Breeds impact the FA profile of meat, proving that genetic selection can improve the nutritional quality of beef [7,21]. For instance, Japanese Black Wagyu contains more MUFAs than other breeds including Holstein, Japanese Brown, Charolais, and Angus [22,23]. When Wagyu steers were crossed with Angus, crossbred steers produced meat higher in MUFAs than Angus when fed a diet high in roughage [24]. Chung et al. [25] hypothesized that Angus steers could equal the MUFA concentrations of Wagyu steers if fed a corn-based diet to a typical U.S. endpoint slaughter weight. Their results showed that beef from Wagyu consistently contained more MUFAs and oleic acid than Angus. Akaushi is one of four Japanese beef breeds that can be called Wagyu [26]. Akaushi is known for its high marbling capabilities and high MUFA content [22,23,27]. Angus is the most common breed in the U.S. and is known for its high feeding efficiency [27,28].

Numerous studies investigated the effects of finishing systems on the FA profile of beef from identical or different cattle breeds [29,30,31,32,33,34]. Most of these studies reported that while diet influences the FA profile of beef, there were strong genetic effects. However, Warren et al. [33] reported that diet had the biggest effect on meat quality.

In the present study, we compared the FA and micronutrient profiles of Red Angus steers (moderate fat breed) and Red Angus x Akaushi crossbreed steers (high fat breed) fed either a complex pasture mixture or a standard grain-based diet. With the increasing demand for healthier beef, there is an urgent need to understand the effects of different diets, breeds, and their interaction on the nutritional profile of beef.

## 2. Materials and Methods

The Michigan State University Institutional Animal Care and Use Committee approved the research protocols for the use of animals and procedures (IACUC #201800155).

### 2.1. Experimental Design

**Figure 1:**
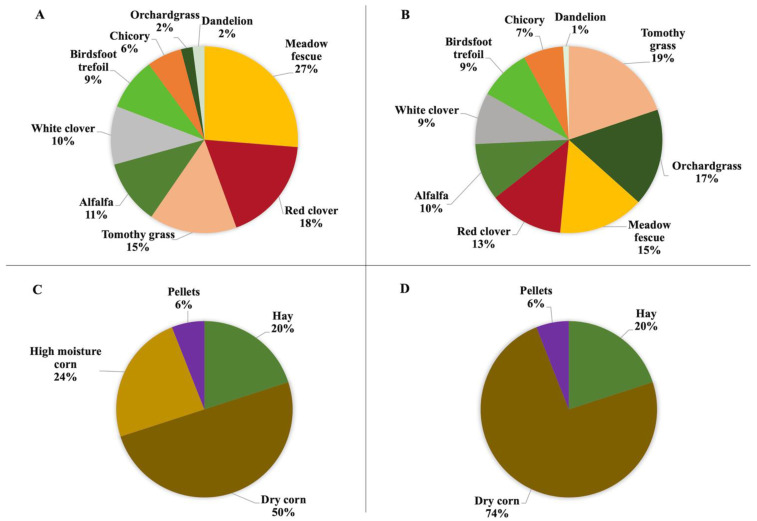
Botanical composition of the diets for 2019 and 2020. (**A**) 2019 GRASS, (**B**) 2020 GRASS, (**C**) 2019 GRAIN, (**D**) 2020 GRAIN. GRASS: complex pasture mixture; GRAIN: conventional grain diet (pellets contained 36% crude protein, hay was made of orchard grass).

The experimental design for this study was reported by Maciel et al. [35]. Briefly, this study was conducted over a period of two years (2019 and 2020) at the Michigan State University Upper Peninsula Research and Extension Center (UPREC) located in Chatham, MI (latitude: 46°20′ N, longitude: 86°55′ W; elevation: 271 m). Sixty steers (*n* = 60) and 44 steers (*n* = 44) (14–20 months old) were randomly allocated using a 2 × 2 factorial experiment in 2019 and 2020, respectively. Two beef breeds were used in this experiment: Red Angus (RA) and Red Angus x Akaushi crossbred (AK). Animals were randomly assigned to one of two finishing systems: a complex pasture mixture (GRASS) or a total mixed feedlot ration (GRAIN). The goal was to have three groups for each finishing system (five animals of each breed in each group). Animals were stratified randomly and assigned to one of the groups for each breed in each finishing system. This design was followed in 2019. As clarified by Maciel et al. [35], less steers were available in 2020 for the GRAIN group due to a low number of male births. Thus, 15 RA and 15 AK were assigned to GRASS, and seven RA and seven AK were assigned to GRAIN for the second year of the study. For GRAIN, two groups were made: one with four animals of each breed, and a second group with three animals of each breed. This was accounted for in the statistical model as described in the Statistical Analysis section. The nutritive value of the experimental diets is displayed in Table 1 and the botanical composition of the diets is shown in Figure 1. For full details about the composition of the diets, see Maciel et al. [35]. An extensive study on the FA and antioxidant profile of the diets used was previously published [36].

### 2.2. Sample Collection

All animals for each year were slaughtered on the same day at a commercial slaughter plant under United Stated Department of Agriculture (USDA) supervision. Body performance and carcass traits were reported by Maciel et al. [35]. Meat samples were collected from the longissimus lumborum (between 11th and 13th rib) on the left side of the carcass. Samples were then transported in a cooler on ice to the Michigan State University Meat Laboratory. A steak was cut into 1 × 1 cm cubes, flash frozen with liquid nitrogen, and stored into Whirl-Pak bags after manually removing all the air. Samples were then stored at −80 °C until further analysis.

### 2.3. Fatty Acid Analysis

All chemicals were purchased from Sigma-Aldrich (St. Louis, MO, USA) unless otherwise noted.

FAs were analyzed following the protocol by Sergin et al. [37]. A microwave-assisted extraction method was used as described by Bronkema et al. [15]. FAs from minced meat samples were extracted using the CEM Mars 6 microwave digestion system equipped with a 24-vessel rotor and a GlassChem vessel set (CEM Corp., Matthews, NC, USA). Briefly, 400 mg of meat was added to a glass vessel with 8 mL of a 4:1 (*v*/*v*) ethyl acetate/methanol solution with 0.1% BHT and was microwaved as follows: 55 °C for 15 min (initial ramp of 2 min at 400 W). Once removed from the microwave, samples were filtered using Whatman qualitative filter paper (grade 597) into a test tube containing 3.5 mL of HPLC-grade water. Samples were then centrifuged at 2500 RPM for 6 min in order to separate the organic and aqueous layers. The top organic layer was transferred into a new test tube and dried under nitrogen gas. The oil was resuspended in a 4:1 (*v*/*v*) dichloromethane/methanol solution with 0.1% BHT. The concentration of each sample was 20 mg of oil/mL.

The method published by Jenkins [38] was adapted for the creation of FA methyl esters (FAMEs). For each sample, 2 mg of suspended oil (100 μL) was aliquoted, dried under nitrogen, and resuspended in toluene with 20 μg of an internal standard (methyl 12-tridecenoate, U-35M, Nu-Chek Prep, Elysian, MN, USA). Subsequently, 2 mL of 0.5 N anhydrous potassium methoxide was added to the samples. Samples were then heated at 50 °C for 10 min. Samples were allowed to cool down to room temperature, and 3 mL of 5% methanolic HCl was added. Samples were heated at 80 °C for 10 min. Once the samples were at room temperature, 2 mL of water and 2 mL of hexane were added. Samples were centrifuged at 2500 RPM for 5 min, and the upper organic layer was removed and dried under nitrogen to obtain FAMEs. The resulting FAMEs were then resuspended in 1 mL of isooctane to obtain a concentration of 2 mg/mL. Samples were transferred to gas chromatography-mass spectrometry (GC-MS) vials with glass inserts. Samples were stored at −20 °C until further analysis.

For the quantification of FAMEs, the PerkinElmer (Waltham, MA, USA) 680/600S GC-MS in the electron impact mode (70 eV) equipped with an Agilent Technologies (Santa Clara, CA, USA) HP-88 column (100 m, 0.25 mm ID, 0.2 μM film thickness) was used. The injection temperature was set at 250 °C, and 1 μL of sample was injected twice (20:1 split) using two different GC parameters (175 °C and 150 °C). The temperature settings were as follows: initial temperature at 80 °C for 4 min; ramp 13 °C/min to 175 °C; hold 27 min; ramp 4 °C/min to 215 °C; hold 35 min, and then an initial temperature at 80 °C for 4 min; ramp 13 °C/min to 150 °C; hold 47 min; ramp 4 °C/min to 215 °C; hold 35 min. A third injection for each sample followed in splitless mode (0.75 min splitless hold time, 40 mL/min flow exiting the vent). This method was modified from Kramer et al. [39] created for improved separation of FA isomers in beef products. Helium was used as the carrier gas at a flow rate of 1 mL/min. The MS data were recorded in full scan mode (mass range of *m*/*z* 70-400 amu). The MS transfer line and ion source temperature were set at 180 °C.

The identification of FAMEs was performed using MassLynx V4.1 SCN 714 (Waters Corporation, Milford, MA, USA). FAs were identified by retention time and EI mass fragmentation in comparison to our reference standard created by using the Supelco 37 Component FAME Mix with mead acid, docosatetraenoic acid, *n*-3 DPA, *n*-6 DPA, and palmitelaidic acid purchased from Cayman Chemical (Ann Arbor, MI, USA). The CLA reference standard UC-59M (Nu-Chek Prep, Elysian, MN, USA) was used to identify CLA isomers. FAs not included in the reference standard were identified according to elution order reported by Kramer et al. [39] and confirmed by the EI mass fragmentation. Beef FAs were reported according to Vahmani et al. [40]. Note that C18:1 4*t* and C18:1 5*t* were below the limit of detection, and C18:2 9*c*,15*t*, C18:2 9*c*,12*t*, and C18:2 9*t*,12*c* were not reported as they were not distinctly separated from the C18:2 11*t*,15*c* peak. Eicosatetraenoic acid (C20:4 *n*-3) was not included in our reference standard and could not be reported. Quantification of FAMEs was performed using a standard curve including the reference and internal standards. The internal standard peak area and analyte peak area relative to the standard curve were used to calculate each FAME concentration. FAs were reported in mg/100 g of beef in this manuscript and in percent of total FAs in the Supplementary Tables.

### 2.4. Vitamin E and Mineral Analysis

Vitamin E and minerals were analyzed by a commercial laboratory (Diagnostic Center for Population and Animal Health, Michigan State University, East Lansing, MI, USA). Vitamin E was measured according to Rettenmaier and Schüep [41]. Briefly, 1 g of beef was mechanically homogenized in 5 mL of water. The solution was frozen to aid in the lysing of cells. After thawing, a measured aliquot was pipetted for extraction. Ethanol was added to precipitate the protein, and hexane was added to extract the vitamins. After centrifugation, a measured portion of the hexane layer was removed and evaporated under reduced pressure in a vortexing chamber (10 min, 35 °C, 300 mBar vacuum). The remaining matter was solubilized in a measured portion of chromatographic mobile phase and placed in vials. A six-point calibration curve was made using the following standard: vitamin E solution (Sigma-Aldrich St. Louis, MO, USA) diluted to working concentrations with ethanol containing BHT followed by serial dilutions (range was 50 μg/mL to 0.2 μg/mL). Samples were analyzed chromatographically using a Waters Acquity system and Water Empower Pro Chromatography Manager software (Water Corporation, Milford, MA, USA). The elution was isocratic using a mobile phase of acetonitrile:methylene chloride:methanol (70:20:10, *v*/*v*/*v*) and a Symmetry C18, 1.7 μm, 2.1 × 50 mm analytical column (Waters Corporation, Milford, MA, USA). The flow rate was set at 0.5mL/min and the detection was done by UV absorption at 292 nm.

Minerals were analyzed according to Wahlen et al. [42]. Beef tissues were dried and digested overnight in a 95 °C oven, using 10× the dry tissue mass of nitric acid. Digested samples were diluted with water to 100× the dried tissue mass. Elemental analysis used an Agilent 7900 Inductivity Coupled Plasma–Mass Spectrometer (ICP-MS) (Agilent Technologies Inc., Santa Clara, CA, USA). An aliquot of each sample and calibration standard were diluted 25-fold with a solution containing 0.5% EDTA and Triton X-100, 1% ammonium hydroxide, 2% butanol, 5 ppb of scandium, and 7.5 ppb of germanium, rhodium, indium, and bismuth as internal standards (Inorganic Ventures, Christiansburg, VA, USA). Concentrations were calibrated using a six-point linear curve of the analyte-internal standard response ratio. Bovine liver and mussel standards (National Institute of Standards and Technology, Gaithersburg, MD, USA) were used as controls. Additionally, a second source calibration check standard was used (Alfa Aesar, Tewksbury, MA, USA).

### 2.5. Statistical Analysis

The statistical analysis was performed using SAS version 9.4 (SAS Institute Inc., Cary, NC, USA). Two-way analysis of variance (ANOVA) was performed. The fixed effects in the statistical model were diet, breed, and the two-way interaction between diet and breed. Random effects included year and pen nested within year, diet, and breed (please see mathematical model below). The experimental unit for this model was each pen. The interaction term was considered first. If not significant, individual effects of diet and breed were considered. Post hoc analysis was performed using the least squares means method. No transformation was needed. Equal variance assumption was satisfied (checked with Levene’s test) which assumed the homogeneity of variance. Results were considered significant at *p* < 0.05. Data are shown as mean ± standard error of mean (SEM).

To correct for the lower number of animals assigned to the GRAIN diet in 2020, some adjustments were made to the statistical model. Year was treated as random effect (and analyzed together instead of each year separately) and each pen was considered the experimental unit leading to combined replicates.

Y_ijkl_ = µ + Y_i_ + P_j_(Y_i_ D_k_ B_l_) + D_k_ + B_l_ + (D_k_ ∗ B_l_) + ℇ_ijkl_

where:

Y = response variable
µ = mean
Y_i_ = year
P_j_(Y_i_ D_k_ B_l_) = pen nested within year, diet, and breed
D_k_ = diet
B_l_ = breed
(D_k_ ∗ B_l_) = interaction between diet and breed
ℇ_ijkl_ = error term

## 3. Results

### 3.1. Saturated, Branched-Chain, and Monounsaturated Fatty Acid Content of Beef

The saturated FA (SFA), branched-chain FA (BCFA), and MUFA content of beef are listed in Table 2. No significant differences by diet, breed, or diet × breed interaction were noted regarding total FA content (*p* > 0.05). Total SFA content did not significantly differ based on diet, breed, or diet × breed. Regarding individual SFAs, C12:0 and C14:0 were significantly higher in beef from AK compared to RA (*p* < 0.05). C16:0 and C18:0 did not show significant differences by diet, breed, or diet × breed interaction (*p* > 0.05). The only diet effects observed were for C15:0 and C19:0; they were significantly higher in GRASS compared to GRAIN (*p* < 0.05).

Regarding total BCFAs, no significant diet, breed, or diet × breed effects were observed (*p* > 0.05). C14:0 *iso*, C15:0 *iso*, C15:0 *anteiso*, and C17:0 *iso* were all significantly higher in beef from GRASS compared to GRAIN (*p* < 0.05).

There were no significant differences observed for the total MUFA content of beef (*p* > 0.05). Total *cis*-MUFAs showed no significant effects (*p* > 0.05). When assessing individual *cis*-MUFAs, C16:1 9*c* was significantly higher in beef from GRAIN compared to GRASS (*p* < 0.01). C16:1 11*c* showed a similar pattern: it was higher in beef from GRAIN compared to GRASS (*p* < 0.01). C18:1 14*c* and C18:1 15*c* were significantly higher in beef from GRASS (*p* < 0.05), while 20:1 11*c* was significantly higher in GRAIN (*p* < 0.05). Total *trans*-MUFAs displayed a significant diet effect; GRASS was higher than GRAIN (*p* < 0.05). C16:1 9*t* was significantly higher in beef from GRASS compared to GRAIN (*p* < 0.001). The same was true for C18:1 11*t* (*p* < 0.05), C18:1 13,14*t* (*p* < 0.01), C18:1 15*t* (*p* < 0.05), and C18:1 16*t* (*p* < 0.05). No significant breed or diet × breed effects were observed (*p* > 0.05).

### 3.2. Polyunsaturated Fatty Acids and Biohydrogenation Intermediate Products

**Figure 2:**
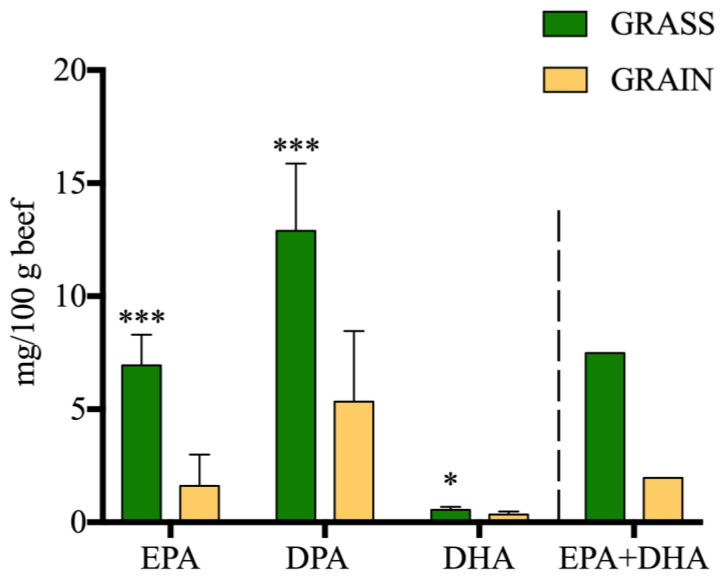
Long-chain *n*-3 PUFA content of beef (mg/100 g beef) by diet. EPA: eicosapentaenoic acid; DPA: *n*-3 docosapentaenoic acid; DHA: docosahexaenoic acid; EPA + DHA: sum of eicosapentaenoic acid and docosahexaenoic acid. ‘*’ denotes statistical significance (‘*’ *p* < 0.05, ‘***’ *p* < 0.001).

The PUFA, CLA, and atypical dienes (AD) content of beef are displayed in Table 3. There were no significant effects on total PUFA content (*p* > 0.05). There was a significant diet effect on total *n*-3 PUFAs, with GRASS containing significantly more *n*-3 PUFAs than GRAIN (34.70 mg per 100g difference; *p* < 0.001). More specifically, ALA, EPA, DPA (*p* < 0.001), and DHA (*p* < 0.05) were all significantly higher in beef from GRASS compared to GRAIN. The sum of EPA + DHA was equal to 7.49 mg per 100 g of meat for beef from GRASS and 1.96 mg per 100 g of meat for beef from GRAIN (Figure 2). The ALA content was 24.63 and 3.14 mg per 100 g of meat for beef from GRASS and GRAIN, respectively. Regarding total *n*-6 PUFAs, there was a significant diet effect with beef from GRAIN containing more *n*-6 PUFAs than GRASS (*p* > 0.01). All individual *n*-6 PUFAs were significantly higher in beef from GRAIN compared to GRASS. The *n*-6:*n*-3 ratio was significantly higher in beef from GRAIN compared to GRASS (8.36 vs. 1.61; *p* < 0.001). Total conjugated linolenic acid (CLnA) (C18:3 9*c*,11*t*,15*t* and C18:3 9*c*,11*t*,15*c*) content was significantly higher in beef from GRASS compared to GRAIN (*p* < 0.05). Total ADs were not significantly different based on diet, breed or diet x breed interaction (*p* > 0.05). C18:2 11*t*,15*t* (*p* < 0.05), C18:2 9*t*,12*t* (*p* < 0.05), and C18:2 11*t*,15*c* (*p* < 0.01) were all higher in beef from GRASS compared to GRAIN. C18:2 9*c*,15*c* was significantly higher in beef from GRAIN compared to GRASS (*p* < 0.01). Total CLA content was significantly higher in beef from GRASS compared to GRAIN (*p* < 0.05). C18:2 11*t*,13*c* (*p* < 0.01) and C18:2 11*t*,13*t* (*p* < 0.05) were significantly higher in beef from GRASS as opposed to GRAIN.

### 3.3. Vitamin E and Mineral Content of Beef

The vitamin E and mineral content of beef is displayed in Table 4. No diet × breed effect was noted for any of the micronutrients. Copper (*p* < 0.01), iron (*p* < 0.001), and molybdenum (*p* < 0.001) were all significantly more abundant in beef from GRASS compared to GRAIN. Manganese was higher in beef from GRAIN vs. GRASS (*p* < 0.05). Selenium showed a breed effect and was present in higher quantity in AK compared to RA (*p* < 0.05). Diet and breed effects were observed for zinc; beef from GRASS contained more zinc than GRAIN (*p* < 0.05), and AK contained more zinc than RA (*p* < 0.01). Finally, vitamin E was significantly higher in beef from GRASS compared to GRAIN (*p* < 0.001).

## 4. Discussion

### 4.1. Saturated Fatty Acids

The U.S. Dietary Guidelines for Americans 2020–2025 recommend limiting saturated fat consumption to 10% of daily caloric intake [43]. This recommendation contributed to the perception that red meat consumption should be limited due to its high SFA content [44]. SFAs promote inflammation and increase low-density lipoprotein (LDL) cholesterol, which is linked to coronary heart diseases. However, this varies by specific SFAs [45]. In the present study, no significant differences in total SFA content were observed between diets and breeds. These findings are in agreement with previous studies reporting no difference in SFA content between grass-fed and grain-fed beef [19,46]. Myristic and palmitic acids have the strongest LDL cholesterol-raising effects compared to other SFAs [47,48]. In the present study, myristic acid was higher in AK compared to RA. May et al. [24] found no significant difference in myristic acid content between Angus and Wagyu beef but the authors reported a higher level of palmitic acid in Angus beef. Stearic acid (C18:0) has a neutral effect on LDL cholesterol [48]. In the present study, no significant differences were observed. Previous studies reported that grass-finishing increased the amount of stearic acid in beef [7,13,49]. May et al. [24] and Chung et al. [25] also reported that RA contained more stearic acid than AK. Malau-Aduli et al. [32] reported significant breed differences in the degree of FA saturation when comparing beef from Jersey and Limousin cows. It was surprising to find no significant differences in LDL-neutral stearic acid between diets and breeds. This may be due to variations in the diets and breeds, but it may also be due to studies reporting FAs in different units. In the present study, when FAs were reported in percent of total FAs, significant differences were seen in stearic acid levels which aligned with the findings of other studies cited above.

### 4.2. Branched-Chain Fatty Acids

BCFAs are mainly SFAs with at least one branching point on their carbon chain that play important roles in the gut health of adults and newborns and are mainly found in ruminant products such as dairy and beef [50]. Additionally, BCFAs might display anti-inflammatory and anti-carcinogenic properties [51]. In the present study, total BCFAs were not significantly different based on diet or breed, but individual BCFAs were higher in beef from GRASS. Information about BCFA content in beef is sparse in the literature, but Picklo et al. [52] reported higher levels of BCFAs in beef from cattle fed a high-forage diet as compared to a low-forage diet. Klopatek et al. [16] also stated that a 100% forage diet would result in higher amounts of BCFAs in beef compared to a standard grain-based diet. The amount of BCFAs in beef is generally inversely related to the forage-to-concentrate ratio [53].

### 4.3. Monounsaturated Fatty Acids

MUFAs are known for their health benefits and their ability to mitigate noncommunicable illnesses such as cardiovascular diseases [45]. Japanese breeds of cattle are known for their marbling and their high MUFA content, so higher levels of MUFAs in beef from AK compared to RA were expected [22,23,24,25]. Maciel et al. [35] also reported higher marbling scores in AK compared to RA. Surprisingly, no significant differences based on diet or breeds were observed in the present study. Oka et al. [54] found that final body weight is negatively correlated with MUFA content, and Maciel et al. [35] found no significant difference in final weight between RA and AK in the same steers used in the present study. This may explain why no differences were seen in total MUFA content between breeds. However, when reporting MUFA content as percent of total FAs, the difference between breeds was significant with beef from AK containing more than RA. MUFAs, in particular oleic acid, contribute to the palatability of beef because of fat softness and their lower melting point [25,55]. We did not observe differences in oleic acid content between the two breeds, which explains the similarities in sensory attributes reported by Maciel et al. [35]. Although AK had greater marbling scores, it did not affect the beef texture and/or flavor as expected [35]. Smith et al. [55] and May et al. [24] reported higher amounts of oleic acid in Wagyu compared to Angus, while Choi et al. [56] reported no significant differences in oleic acid between Angus and Wagyu crossbreeds.

Grain-finished beef is expected to be higher in total MUFAs as reported in previous studies [13,16,57]. We did not observe differences between diets for total MUFAs, but some individual *cis*-MUFAs (C16:1) were higher in beef from GRAIN compared to GRASS. Regarding benefits for human health, *cis*-MUFAs (especially oleic acid) are of interest because of their LDL cholesterol-lowering potential [47]. In this study, we did not find significant differences in oleic acid content between diets and breeds, although numerous studies found that oleic acid is usually higher in grain-fed beef compared to GFB [13,16,57,58].

In this study, grass-finishing increased the amount of *trans*-MUFAs in beef. Klopatek et al. [16] reported higher levels of *trans*-MUFAs in grain-finished beef compared to GFB, while Nuernberg et al. [58] observed higher concentrations of total *trans*-C18:1 in GFB compared to cattle fed a concentrate diet. The effects of ruminal *trans*-unsaturated FAs on human health remain uncertain. While the association of ruminal *trans*-FAs with coronary heart diseases remains unclear [59], other studies reported potential negative health effects [60,61]. However, the health effects of *trans*-MUFAs are isomer specific [62,63]. For example, vaccenic acid (C18:1 *t*11) reduces plasma triglycerides and improves immune functions while C18:1 *t*9 and *t*10 have been associated with negative health effects [62,64]. Mapiye et al. [62] reported that finishing cattle on grass can increase vaccenic acid concentrations relative to C18:1 *t*10. In the present study, vaccenic acid was significantly higher in beef from GRASS compared to GRAIN. Our results indicate that while grass-finishing increased the total *trans*-MUFA content of beef, this difference was mainly due to an increase in beneficial vaccenic acid.

### 4.4. Polyunsaturated Fatty Acids

Consumer interest in health foods continues to increase, and researchers and producers are investigating ways to improve the nutritional quality of beef to contribute to reducing noncommunicable diseases in humans [17,65]. Scollan et al. [17] reported that increasing the *n*-3 PUFA and CLA contents of beef while reducing SFAs and the *n*-6:*n*-3 ratio are important priorities. The Western diet is usually high in *n*-6 PUFAs and deficient in beneficial *n*-3 PUFAs [66]. Long-chain *n*-3 PUFAs are thought to have anti-inflammatory properties while *n*-6 PUFAs do not [67]. GFB contains *n*-3 PUFAs but mainly as ALA which is not efficiently converted into beneficial long-chain *n*-3 PUFAs such as EPA and DHA [68,69]. Most health benefits are linked to EPA and DHA which are related to healthier cardiovascular function [70,71].

All *n*-6 PUFAs were higher in beef from GRAIN compared to GRASS, similar to the results reported in previous studies [7,16,34], and no breed effect was observed. Chung et al. [25] reported that LA (C18:2 *n*-6) content varied based on breed and diet when comparing Angus and Wagyu fed either corn or hay. Angus was higher in LA compared to Wagyu when fed corn but was lower in LA when fed hay. GFB is known to be higher in *n*-3 PUFAs compared to conventional grain-finished beef [7,16,18,72,73]. The European Union considers a “source of omega-3 fatty acids” a food that contains at least 0.3 g of ALA per 100 g serving or at least 40 mg of EPA + DHA per 100 g serving, and a “good source of omega-3 fatty acids” a food that contains at least 0.6 g of ALA per 100 g serving or at least 80 mg of EPA + DHA per 100 g serving [74]. Based on European standards, our GFB would not qualify as a “source of omega-3 fatty acids”. Even though DPA is not investigated as much as EPA and DHA for its health benefits, it has been linked to improved cognitive functions, lower blood triglycerides, lower cholesterol, lower inflammation, and lower risks of coronary heart diseases [75]. Our results indicate that even if GFB does not qualify as a “source” of long-chain *n*-3 PUFAs, it can still contribute to the daily intake for individuals who do not have access to marine foods [76]. We did not observe significant differences in *n*-3 PUFAs between breeds in the current study. Liu et al. [77] found no significant difference in total *n*-3 PUFA content between crossbred Angus x Simmental beef and Wagyu x Simmental beef, while Chung et al. [25] reported that Wagyu had higher ALA content than Angus when fed to the same endpoint. Warren et al. [34] noted subtle differences in PUFA content by breed.

A low *n*-6:*n*-3 ratio is an important factor to prevent noncommunicable diseases [67,78]. An optimal ratio for human health is suggested around 1:1 as found in the meat of wild animals or traditional human diets [66,67]. In this study, the *n*-6:*n*-3 ratio only differed based on diet. Beef from GRASS had a ratio of 1.61:1 while beef from GRAIN had a ratio of 8.36:1. Grass-finishing was expected to raise the *n*-3 PUFA content of beef, consequently lowering the *n*-6:*n*-3 ratio [16,18,46,72,79].

### 4.5. Biohydrogenation Intermediate Products

PUFAs are toxic to many rumen bacteria, which is why they undergo biohydrogenation in the rumen. LA and ALA undergo extensive biohydrogenation (70–95% and 85–100%, respectively) [80]. When LA and ALA go through biohydrogenation in the rumen, multiple intermediate compounds are produced including CLnA, CLA, and AD [81]. In the present study, beef fed GRASS contained significantly more CLnA than beef fed GRAIN. This was expected since most of the ALA found in GFB comes from fresh forage [79]. Regarding AD, Klopatek et al. [16] reported similar results that the present study and highlighted that health effects of AD remain unknown. CLA and its precursor *trans*-vaccenic acid (TVA) are thought to have health benefits including regulating insulin resistance and blood pressure and improving lipid metabolism [82,83,84]. CLA is also purported to have anticarcinogenic effects [85]. In the present study, total CLA content was higher in beef from GRASS than beef from GRAIN, which was also reported in other studies [7,57]. We did not find any significant differences in the concentration of biohydrogenation intermediate products based on breed. This result was surprising since it was previously reported that Wagyu contained more CLA than European and British crossbred cattle [86] and that Wagyu contained more CLA per 100 g of meat than Limousin cattle [85]. The authors attributed these changes mainly to the difference of total fat content.

### 4.6. Vitamin E and Minerals

The higher levels of micronutrients in GFB found in the present study are supported by the literature and may be due to GFB being leaner than grain-finished beef [13,31,33,57]. Vitamin E acts as an antioxidant and protects cells against free radicals, but it can also extend the shelf-life of meat [79]. De la Fuente et al. [87] reported significantly higher levels of vitamin E in GFB compared to conventional beef, enough to protect from oxidation. Warren et al. [33] previously reported that diet had the biggest impact on meat quality and found higher vitamin E levels in GFB compared to beef finished on a concentrate diet. Horcada et al. [31] showed that GFB had a higher PUFA and antioxidant content compared to grain-finished beef, and that the higher antioxidant levels (mainly as vitamin E) found in GFB resulted in the stability of the FAs. This led the authors to recommend the consumption of GFB to benefit human health. There is a lack of information about micronutrient content of Angus vs. Akaushi beef in the literature. Li et al. [88] published a study comparing the mineral content of Quichuan and Wagyu x Quichuan cattle liver. Their results showed that the crossbred Wagyu x Quichuan cattle liver contained significantly more iron, zinc, selenium, and manganese than the Quichuan cattle. These findings indicate that crossbreeding with Wagyu can potentially increase the mineral content of liver and meat.

## 5. Conclusions

Our results indicate that the diet effect is more dominant than the breed in this study, meaning that the cattle diet remains the most efficient way to improve the nutritional profile of beef. This is novel since most studies in the literature report strong genetic effects. Grass-finishing improved bioactive compounds of interest when compared to grain-finishing. These include *n*-3 PUFAs, the *n*-6:*n*-3 ratio, CLnA, CLA, *trans*-vaccenic acid, copper, iron, zinc, and vitamin E. To our surprise, we did not observe many significant differences by breed and diet × breed effects. Most breed effects were observed for SFAs; AK was higher in some SFAs such as lauric and myristic acids that do not favor human health [47]. Surprisingly, we did not find significant differences in MUFA content based on diet or breed. AK beef was higher in micronutrients such as selenium and zinc. Crossbreeding Akaushi with Red Angus did not significantly improve the nutritional profile of beef.

Our study contains some limitations; we did have a control group that was 100% Red Angus, but we did not have another control group that was 100% Akaushi. Additionally, we did not feed our animals to multiple endpoints (i.e., Japanese vs. U.S.) which may explain why we did not observe any diet × breed effects. The literature does not contain much information on the specific breeds and diets that were used in the present study, making direct comparisons difficult. However, we believe that our work fills a gap in the literature and adds important information to the current knowledge about grass-finishing (especially on diverse pastures) and improving the nutritional quality of beef for human health. To our knowledge, this is the first paper to report a comprehensive list of FAs (including BCFAs, ADs, CLA and CLnA isomers) to compare two popular breeds and two finishing systems. More research is needed to better understand how to combine breeds and finishing systems to improve the nutrient profile of beef to favor human health.

## Figures and Tables

**Table 1:** Nutritive value of experimental diets (% of total fatty acids) ^1^.

	2019		2020	
	Grass	Grain	Grass	Grain
C16:0	13.73	14.55	14.48	12.74
C18:0	1.86	1.89	1.61	1.43
C18:1 *n*-7	0.23	0.53	0.78	0.49
C18:1 *n*-9	4.10	22.45	2.36	21.99
C18:2 *n*-6 (LA) ^2^	13.25	53.97	14.82	59.33
C18:3 *n*-3 (ALA) ^3^	62.35	5.24	60.93	2.87
∑ SFA ^4^	18.92	17.56	19.74	15.08
∑ MUFA ^5^	5.41	23.22	4.52	22.72
∑ PUFA ^6^	75.66	59.22	75.75	62.2
∑ *n*-6 ^7^	13.25	53.98	14.82	59.33
∑ *n*-3 ^8^	62.35	5.24	60.93	2.87
*n*-6:*n*-3 ratio ^9^	0.22	10.77	0.25	21.63
Dry matter (DM) (%)	20.54	76.11	22.11	85.32
Ash *	7.13	4.59	6.14	3.17
Crude protein *	11.54	9.83	15.03	9.38
Neutral detergent fiber (NDF) *	52.21	21.22	51.49	20.71
Acid detergent fiber (ADF) *	34.99	10.24	31.98	9.87
Energy (cal/g)	4407.44	4223.98	4516.28	4328.79

^1^ Reported according to Krusinski et al. [36]; Values expressed as means; ^2^ LA: linoleic acid; ^3^ ALA: alpha-linoleic acid; ^4^ ∑ SFA = all saturated FAs 10:0 through 24:0 (even and odd); ^5^ ∑ MUFA = all monounsaturated FAs 16:1-18:1; ^6^ ∑ PUFA = LA + ALA; ^7^ ∑ *n*-6 = LA; ^8^ ∑ *n*-3 = ALA; ^9^ *n*-6:*n*-3 ratio = ∑ *n*-6/∑ *n*-3; * expressed as %DM.

**Table 2:** Mean concentrations of saturated and monounsaturated fatty acids in beef by diet, breed, and diet × breed interaction (mg per 100 g meat) ^1^.

Fatty Acid	Diet (D)		Breed (B)		Significance of ANOVA ^4^		
	GRASS	GRAIN	RA ^2^	AK ^3^	D	B	D × B
∑ SFA ^5^	892.85 ± 514.91	1118.49 ± 521.82	856.66 ± 518.08	1154.67 ± 518.08	NS	NS	NS
C10:0	1.54 ± 0.27	1.54 ± 0.30	1.40 ± 0.27	1.68 ± 0.27	NS	NS	NS
C12:0	1.23 ± 0.67	1.32 ± 0.68	0.97 ± 0.67	1.58 ± 0.67	NS	*	NS
C13:0	0.13 ± 0.06	0.10 ± 0.06	0.10 ± 0.06	0.12 ± 0.06	NS	NS	NS
C14:0	40.44 ± 25.03	67.50 ± 25.60	41.06 ± 25.29	66.87 ± 25.29	NS	*	NS
C15:0	9.92 ± 5.18	4.15 ± 5.27	6.04 ± 5.22	8.03 ± 5.22	*	NS	NS
C16:0	507.08 ± 309.05	767.23 ± 312.85	540.29 ± 310.79	734.02 ± 310.79	NS	NS	NS
C17:0	26.57 ± 14.31	12.17 ± 14.56	16.88 ± 14.33	21.85 ± 14.33	NS	NS	NS
C18:0	302.69 ± 158.95	263.28 ± 161.50	248.01 ± 160.12	317.97 ± 160.12	NS	NS	NS
C19:0	1.32 ± 0.83	0.50 ± 0.84	0.80 ± 0.83	1.01 ± 0.83	*	NS	NS
C20:0	1.27 ± 0.68	0.77 ± 0.70	0.83 ± 0.69	1.20 ± 0.69	NS	NS	NS
C22:0	0.68 ± 0.14	0.75 ± 0.14	0.68 ± 0.14	0.75 ± 0.14	NS	NS	NS
∑ BCFA ^6^	34.37 ± 15.25	18.00 ± 15.60	21.69 ± 15.41	30.68 ± 15.41	NS	NS	NS
C14:0 *iso*	0.76 ± 0.38	0.17 ± 0.39	0.38 ± 0.39	0.56 ± 0.38	*	NS	NS
C15:0 *iso*	3.65 ± 1.94	1.20 ± 1.98	2.06 ± 1.96	2.79 ± 1.96	*	NS	NS
C15:0 *anteiso*	2.60 ± 1.32	0.63 ± 1.35	1.38 ± 1.33	1.86 ± 1.33	*	NS	NS
C16:0 *iso*	2.79 ± 1.88	2.39 ± 1.90	2.07 ± 1.89	3.11 ± 1.89	NS	NS	NS
C17:0 *iso*	10.75 ± 3.56	3.84 ± 3.68	6.27 ± 3.61	8.32 ± 3.61	*	NS	NS
C17:0 *anteiso*	11.91 ± 4.88	7.69 ± 5.03	7.95 ± 4.95	11.65 ± 4.95	NS	NS	NS
C18:0 *iso*	1.89 ± 1.31	2.11 ± 1.33	1.60 ± 1.32	2.40 ± 1.32	NS	NS	NS
∑ MUFA ^7^	893.35 ± 459.75	1109.48 ± 468.21	833.75 ± 463.63	1169.07 ± 463.63	NS	NS	NS
∑ *c*MUFA ^8^	819.76 ± 434.62	1093.68 ± 442.53	792.82 ± 438.25	1160.62 ± 438.25	NS	NS	NS
C14:1 9*c*	7.85 ± 7.10	20.80 ± 7.24	11.16 ± 7.16	17.49 ± 7.16	**	NS	NS
C16:1 9*c*	75.85 ± 28.21	152.90 ± 29.77	92.42 ± 28.94	136.33 ± 28.94	**	NS	NS
C16:1 10*c*	3.50 ± 0.60	5.81 ± 0.70	3.86 ± 0.65	5.45 ± 0.65	*	NS	NS
C16:1 11*c*	1.68 ± 0.61	4.32 ± 0.66	2.43 ± 0.63	3.57 ± 0.63	**	NS	NS
C17:1 9*c*	11.13 ± 5.57	10.59 ± 5.67	9.42 ± 5.61	12.30 ± 5.61	NS	NS	NS
C18:1 9*c*	682.04 ± 379.00	849.55 ± 385.08	637.07 ± 381.78	849.51 ± 381.78	NS	NS	NS
C18:1 11*c*	22.88 ± 10.87	30.89 ± 11.08	22.50 ± 10.96	31.27 ± 10.96	NS	NS	NS
C18:1 12*c*	3.41 ± 1.34	4.09 ± 1.41	3.31 ± 1.37	4.20 ± 1.37	NS	NS	NS
C18:1 13*c*	3.99 ± 1.42	6.16 ± 1.49	4.00 ± 1.45	6.15 ± 1.45	NS	NS	NS
C18:1 14*c*	0.67 ± 0.08	0.29 ± 0.09	0.44 ± 0.08	0.52 ± 0.08	*	NS	NS
C18:1 15*c*	1.38 ± 0.54	0.67 ± 0.56	0.81 ± 0.55	1.24 ± 0.55	*	NS	NS
C20:1 9*c*	1.86 ± 0.33	2.17 ± 0.38	1.74 ± 0.35	2.29 ± 0.35	NS	NS	NS
C20:1 11*c*	3.51 ± 0.66	6.08 ± 0.77	3.97 ± 0.71	5.61 ± 0.71	*	NS	NS
∑ *t*MUFA ^9^	73.59 ± 25.39	17.05 ± 26.44	41.56 ± 25.59	49.08 ± 25.59	*	NS	NS
C16:1 9*t*	3.49 ± 0.18	1.30 ± 0.21	2.30 ± 0.20	2.48 ± 0.20	***	NS	NS
C16:1 10-12*t*	9.01 ± 2.97	4.83 ± 3.08	6.07 ± 3.02	7.78 ± 3.02	NS	NS	NS
C18:1 6-8*t*	1.79 ± 0.40	0.96 ± 0.44	1.25 ± 0.42	1.50 ± 0.42	NS	NS	NS
C18:1 9*t*	2.33 ± 0.55	2.04 ± 0.59	1.96 ± 0.57	2.42 ± 0.57	NS	NS	NS
C18:1 10*t*	2.24 ± 0.65	1.48 ± 0.69	1.68 ± 0.66	2.04 ± 0.66	NS	NS	NS
C18:1 11*t*	34.52 ± 13.35	1.10 ± 13.99	16.43 ± 13.34	19.19 ± 13.34	*	NS	NS
C18:1 12*t*	4.47 ± 2.28	2.09 ± 2.33	2.74 ± 2.30	3.82 ± 2.30	NS	NS	NS
C18:1 13,14*t*	8.57 ± 2.73	1.45 ± 2.87	4.84 ± 2.79	5.19 ± 2.79	**	NS	NS
C18:1 15*t*	2.53 ± 0.46	0.91 ± 0.52	1.72 ± 0.49	1.72 ± 0.49	*	NS	NS
C18:1 16*t*	4.63 ± 1.99	1.14 ± 2.07	2.69 ± 2.02	3.08 ± 2.02	*	NS	NS
∑ FA ^10^	1962.72 ± 1033.46	2376.72 ± 1049.38	1840.80 ± 1040.75	2498.64 ± 1040.75	NS	NS	NS

^1^ Values reported as means ± SEM (standard error of mean). ^2^ RA; Red Angus, ^3^ AK; Red Angus x Akaushi, ^4^ NS; not significant; *p* > 0.05; * *p* < 0.05; ** *p* < 0.01; *** *p* < 0.001. ^5^ ∑ SFA = all saturated FAs (10:0, 12:0, 13:0, 14:0, 15:0, 16:0, 17:0, 18:0, 19:0, 20:0, 22:0); ^6^ ∑ BCFA = sum of all branched chain FAs (*iso*14:0, *iso*15:0, *anteiso*15:0, *iso*16:0, *iso*17:0, *anteiso*17:0, *iso*18:0); ^7^ ∑ MUFA = all monounsaturated FAs (14:1, 16:1, 17:1, 18:1, 20:1); ^8^ ∑ *c*MUFA = 14:1, 17:1, sum of *c*16:1, *c*18:1, and *c*20:1; ^9^ ∑ *t*MUFA = sum of *t*16:1 and *t*18:1; ^10^ ∑ FA = sum of all FAs.

**Table 3:** Mean concentrations of polyunsaturated fatty acids in beef by diet, breed, and diet × breed interaction (mg per 100 g meat) ^1^.

Fatty Acid	Diet (D)		Breed (B)		Significance of ANOVA ^4^		
	GRASS	GRAIN	RA ^2^	AK ^3^	D	B	D × B
∑ PUFA ^5^	113.13 ± 32.84	112.12 ± 33.15	108.30 ± 32.98	116.95 ± 32.98	NS	NS	NS
∑ *n*-3 ^6^	45.34 ± 12.02	10.64 ± 12.20	27.47 ± 11.94	28.49 ± 11.94	***	NS	NS
C18:3 *n*-3 (ALA) ^7^	24.63 ± 7.80	3.14 ± 7.93	13.18 ± 7.82	14.59 ± 7.82	***	NS	NS
C20:3 *n*-3	0.31 ± 0.09	0.07 ± 0.09	0.16 ± 0.09	0.22 ± 0.09	**	NS	NS
C20:5 *n*-3 (EPA) ^8^	6.94 ± 1.36	1.61 ± 1.38	4.49 ± 1.35	4.07 ± 1.35	***	NS	NS
C22:5 *n*-3 (DPA) ^9^	12.90 ± 2.97	5.34 ± 3.12	9.14 ± 2.93	9.10 ± 2.93	***	NS	NS
C22:6 *n*-3 (DHA) ^10^	0.55 ± 0.13	0.35 ± 0.13	0.45 ± 0.13	0.44 ± 0.13	*	NS	NS
∑ *n*-6 ^11^	66.91 ± 20.44	101.36 ± 20.69	80.31 ± 20.55	87.96 ± 20.55	**	NS	NS
C18:2 *n*-6 (LA) ^12^	48.07 ± 16.84	69.08 ± 17.06	54.30 ± 16.94	62.86 ± 16.94	*	NS	NS
C18:3 *n*-6	0.29 ± 0.02	0.38 ± 0.03	0.33 ± 0.03	0.35 ± 0.03	*	NS	NS
C20:2 *n*-6	0.38 ± 0.08	0.58 ± 0.09	0.45 ± 0.08	0.52 ± 0.08	*	NS	NS
C20:3 *n*-6	3.20 ± 1.09	5.63 ± 1.11	4.34 ± 1.10	4.49 ± 1.10	***	NS	NS
C20:4 *n*-6	13.44 ± 2.88	21.02 ± 2.91	17.68 ± 2.89	16.79 ± 2.89	***	NS	NS
C22:4 *n*-6	1.53 ± 0.51	4.68 ± 0.54	3.23 ± 0.50	2.98 ± 0.50	***	NS	NS
*n*-6:*n*-3 ratio ^13^	1.61 ± 0.39	8.36 ± 0.41	5.04 ± 0.39	4.92 ± 0.39	***	NS	NS
C20:3 *n*-9	0.88 ± 0.17	1.10 ± 0.17	0.98 ± 0.17	0.98 ± 0.17	NS	NS	NS
∑ CLnA ^14^	0.38 ± 0.15	0.26 ± 0.15	0.29 ± 0.15	0.34 ± 0.15	*	NS	NS
C18:3 9*c*,11*t*,15*t*	0.12 ± 0.02	0.03 ± 0.02	0.07 ± 0.02	0.08 ± 0.02	*	NS	NS
C18:3 9*c*,11*t*,15*c*	0.26 ± 0.14	0.23 ± 0.14	0.23 ± 0.14	0.27 ± 0.14	NS	NS	NS
∑ AD ^15^	19.86 ± 9.06	11.70 ± 9.22	13.60 ± 9.14	17.95 ± 9.14	NS	NS	NS
C18:2 11*t*,15*t*	5.01 ± 2.01	2.11 ± 2.07	3.02 ± 2.04	4.10 ± 2.04	*	NS	NS
C18:2 9*t*,12*t*	0.85 ± 0.14	0.17 ± 0.16	0.50 ± 0.14	0.52 ± 0.14	*	NS	NS
C18:2 9*c*,14*t*/9*c*,13*t*	2.72 ± 1.05	1.76 ± 1.08	1.89 ± 1.06	2.59 ± 1.06	NS	NS	NS
C18:2 11*t*,15*c*	5.39 ± 1.96	0.40 ± 2.04	2.58 ± 1.97	3.21 ± 1.97	**	NS	NS
C18:2 9*c*,16*t*	4.28 ± 3.19	4.37 ± 3.20	3.71 ± 3.19	4.94 ± 3.19	NS	NS	NS
C18:2 9*c*,15*c*	1.18 ± 0.53	2.80 ± 0.55	1.70 ± 0.54	2.28 ± 0.54	**	NS	NS
C18:2 12*c*,15*c*	0.42 ± 0.30	0.25 ± 0.30	0.28 ± 0.30	0.39 ± 0.30	NS	NS	NS
∑ CLA ^16^	5.70 ± 0.72	2.70 ± 0.84	3.78 ± 0.78	4.62 ± 0.78	*	NS	NS
C18:2 9*c*,11*t*/9*c*,7*t*	4.54 ± 0.67	2.18 ± 0.78	2.97 ± 0.70	3.75 ± 0.70	NS	NS	NS
C18:2 11*t*,13*c*	0.47 ± 0.07	0.18 ± 0.08	0.31 ± 0.07	0.34 ± 0.07	**	NS	NS
C18:2 11*t*,13*t*	0.36 ± 0.14	0.20 ± 0.14	0.28 ± 0.14	0.28 ± 0.14	*	NS	NS
C18:2 *t,t*	0.33 ± 0.11	0.25 ± 0.11	0.28 ± 0.11	0.30 ± 0.11	NS	NS	NS

^1^ Values reported as means ± SEM (standard error of mean). ^2^ RA; Red Angus, ^3^ AK; Red Angus x Akaushi, ^4^ NS; not significant; *p* > 0.05; * *p* < 0.05; ** *p* < 0.01; *** *p* < 0.001. ^5^ ∑ PUFA = LA + ALA + GLA + Eicosadienoic + Eicosatrienoic + DGLA + Mead + Arachidonic + EPA + DTA + DPA *n*-3 + DHA; ^6^ ∑ *n*-3 = ALA + EPA + DHA + DPA *n*-3 + Eicosatrienoic; ^7^ ALA; alpha-linolenic acid, ^8^ EPA; eicosapentaenoic acid, ^9^ DPA; *n*-3 docosapentaenoic acid, ^10^ DHA; docosahexaenoic acid; ^11^ ∑ *n*-6 = LA + GLA + Eicosadienoic + DGLA + Arachidonic + DTA; ^12^ LA; linoleic acid; ^13^ *n*-6:*n*-3 ratio = ∑ *n*-6/∑ *n*-3; ^14^ ∑ CLnA = sum of conjugated linolenic acid isomers (*c*9, *t*11, *t*15 18:3 + *c*9, *t*11, *c*15 18:3); ^15^ ∑ Atypical Dienes (AD) = sum of non-conjugated linoleic acid isomers (*t*11, *t*15 18:2 + *t*9, *t*12 18:2 + *c*9, *t*14/*c*9, *t*13 18:2 + *t*11, *c*15 18:2 + *c*9, *t*16 18:2 + *c*9, *c*15 18:2 + *c*12, *c*15 18:2); ^16^ ∑ CLA = sum of conjugated linoleic acid isomers (*c*9, *t*11/*t*7, *c*9 18:2 + *t*11, *c*13 18:2 + *t*11, *t*13 18:2 + *t,t* 18:2).

**Table 4:** Diet, breed, and interaction effects on micronutrient content of beef (mg per 100 g of beef) ^1^.

Micronutrient	Diet (D)		Breed (B)		Significance of ANOVA ^4^		
	GRASS	GRAIN	RA ^2^	AK ^3^	D	B	D × B
Copper	0.17 ± 0.01	0.08 ± 0.01	0.12 ± 0.01	0.14 ± 0.01	**	NS	NS
Iron	6.76 ± 0.10	4.85 ± 0.12	5.90 ± 0.15	5.71 ± 0.15	***	NS	NS
Manganese	0.01 ± 0.00	0.02 ± 0.00	0.02 ± 0.00	0.02 ± 0.00	*	NS	NS
Molybdenum	0.02 ± 0.00	0.00 ± 0.00	0.01 ± 0.00	0.01 ± 0.00	***	NS	NS
Selenium	0.05 ± 0.00	0.05 ± 0.00	0.04 ± 0.00	0.05 ± 0.00	NS	*	NS
Zinc	13.61 ± 0.36	11.83 ± 0.32	12.20 ± 0.26	13.47 ± 0.26	*	**	NS
Vitamin E	3.42 ± 0.13	1.69 ± 0.06	2.62 ± 0.18	2.50 ± 0.18	***	NS	NS

^1^ Values reported as means ± SEM (standard error of mean); ^2^ RA; Red Angus, ^3^ AK; Red Angus x Akaushi, ^4^ NS, not significant, *p* > 0.05; * *p* < 0.05; ** *p* < 0.01; *** *p* < 0.001.

## Data Availability

The original contributions presented in the study are included in the article/Supplementary Material. Further inquiries can be directed to the corresponding author.

## Supplementary Materials

The following supporting information can be downloaded at: https://www.mdpi.com/article/10.3390/foods11213451/s1, Table S1: Mean concentrations of saturated and monounsaturated fatty acids in beef by diet, breed, and diet × breed interaction (% of total fatty acids); Table S2: Mean concentrations of polyunsaturated fatty acids in beef by diet, breed, and diet × breed interaction (% of total fatty acids).
