# Supplementary material for: Fatty Acid and Micronutrient Profile of Longissimus Lumborum from Red Angus and Red Angus x Akaushi Cattle Finished on Grass or Grain
Source: Foods. 2022 Oct 31;11(21):3451. doi: 10.3390/foods11213451 (PMC9656263; doi:10.3390/foods11213451)
Supplement: Supplementary file 1 [file foods-11-03451-s001.zip › foods-1974792-supplementary.pdf]

Table S1. Mean concentrations of saturated and monounsaturated fatty acids in beef by diet, breed, and diet × breed interaction (% of total fatty acids)[1]

Fatty acid	*Diet (D)*		*Breed (B)*		*Significance of ANOVA*[4]		
	**GRASS**	**GRAIN**	**RA**[2]	**AK**[3]	**D**	**B**	**D × B**
**∑ SFA**[5]	43.86 ± 2.84	45.68 ± 2.86	45.12 ± 2.84	44.41 ± 2.84	NS	NS	NS
C10:0	0.11 ± 0.03	0.08 ± 0.03	0.10 ± 0.03	0.09 ± 0.03	NS	NS	NS
C12:0	0.06 ± 0.00	0.06 ± 0.00	0.05 ± 0.00	0.06 ± 0.00	NS	***	NS
C13:0	0.01 ± 0.00	0.00 ± 0.00	0.01 ± 0.00	0.01 ± 0.00	*	NS	NS
C14:0	1.86 ± 0.08	2.52 ± 0.09	2.02 ± 0.08	2.36 ± 0.08	***	***	NS
C15:0	0.38 ± 0.06	0.18 ± 0.06	0.26 ± 0.06	0.29 ± 0.06	***	NS	NS
C16:0	25.87 ± 1.94	31.24 ± 1.98	28.67 ± 1.89	28.44 ± 1.89	**	NS	NS
C17:0	1.06 ± 0.23	0.52 ± 0.23	0.79 ± 0.22	0.80 ± 0.22	***	NS	NS
C18:0	14.36 ± 0.70	10.92 ± 0.71	13.06 ± 0.70	12.21 ± 0.70	***	*	NS
C19:0	0.05 ± 0.02	0.02 ± 0.02	0.04 ± 0.02	0.03 ± 0.02	***	NS	NS
C20:0	0.05 ± 0.01	0.03 ± 0.01	0.04 ± 0.01	0.04 ± 0.01	***	NS	NS
C22:0	0.05 ± 0.01	0.05 ± 0.01	0.05 ± 0.01	0.04 ± 0.01	NS	NS	NS
**∑ BCFA**[6]	1.53 ± 0.05	0.75 ± 0.06	1.08 ± 0.05	1.19 ± 0.05	***	NS	NS
C14:0 *iso*	0.03 ± 0.00	0.01 ± 0.00	0.01 ± 0.00	0.02 ± 0.00	***	NS	NS
C15:0 *iso*	0.14 ± 0.04	0.08 ± 0.04	0.10 ± 0.04	0.11 ± 0.04	NS	NS	NS
C15:0 *anteiso*	0.10 ± 0.02	0.03 ± 0.02	0.06 ± 0.02	0.07 ± 0.02	***	NS	NS
C16:0 *iso*	0.11 ± 0.04	0.08 ± 0.04	0.09 ± 0.04	0.09 ± 0.04	**	NS	NS
C17:0 *iso*	0.54 ± 0.07	0.19 ± 0.07	0.35 ± 0.07	0.38 ± 0.07	***	NS	NS
C17:0 *anteiso*	0.54 ± 0.04	0.30 ± 0.04	0.40 ± 0.04	0.45 ± 0.04	***	NS	NS
C18:0 *iso*	0.07 ± 0.03	0.07 ± 0.03	0.07 ± 0.03	0.07 ± 0.03	NS	NS	NS
**∑ MUFA**[7]	44.59 ± 1.21	46.17 ± 1.28	44.62 ± 1.14	46.14 ± 1.14	NS	*	NS
**∑ *c*MUFA**[8]	41.21 ± 0.89	45.07 ± 0.98	42.39 ± 0.79	43.90 ± 0.79	*	*	NS
C14:1 9*c*	0.35 ± 0.08	0.75 ± 0.08	0.50 ± 0.08	0.60 ± 0.08	***	*	NS
C16:1 9*c*	4.42 ± 1.28	6.62 ± 1.28	5.26 ± 1.28	5.78 ± 1.28	***	*	NS
C16:1 10*c*	0.24 ± 0.10	0.25 ± 0.10	0.23 ± 0.10	0.26 ± 0.10	NS	*	NS
C16:1 11*c*	0.10 ± 0.04	0.17 ± 0.04	0.12 ± 0.04	0.14 ± 0.04	***	**	NS
C17:1 9*c*	0.53 ± 0.01	0.44 ± 0.02	0.47 ± 0.01	0.50 ± 0.01	**	NS	NS
C18:1 9*c*	33.46 ± 1.06	34.72 ± 1.11	33.72 ± 1.02	34.47 ± 1.02	NS	NS	NS
C18:1 11*c*	1.24 ± 0.09	1.38 ± 0.10	1.30 ± 0.09	1.32 ± 0.09	NS	NS	NS
C18:1 12*c*	0.17 ± 0.03	0.15 ± 0.03	0.16 ± 0.03	0.16 ± 0.03	NS	NS	NS
C18:1 13*c*	0.22 ± 0.06	0.27 ± 0.06	0.23 ± 0.06	0.26 ± 0.06	**	NS	NS
C18:1 14*c*	0.04 ± 0.02	0.02 ± 0.02	0.03 ± 0.02	0.03 ± 0.02	***	NS	NS
C18:1 15*c*	0.07 ± 0.01	0.04 ± 0.01	0.05 ± 0.01	0.06 ± 0.01	**	NS	*
C20:1 9*c*	0.11 ± 0.04	0.10 ± 0.04	0.10 ± 0.04	0.11 ± 0.04	NS	NS	*
C20:1 11*c*	0.26 ± 0.15	0.29 ± 0.15	0.27 ± 0.15	0.28 ± 0.15	NS	NS	NS

**∑ *t*MUFA[9]**	3.37 ± 0.36	1.04 ± 0.37	2.20 ± 0.37	2.22 ± 0.37	***	NS	NS
C16:1 9*t*	0.30 ± 0.10	0.09 ± 0.10	0.20 ± 0.10	0.19 ± 0.10	**	NS	NS
C16:1 10-12*t*	0.50 ± 0.08	0.26 ± 0.08	0.35 ± 0.08	0.37 ± 0.08	***	NS	NS
C18:1 6-8*t*	0.10 ± 0.03	0.05 ± 0.03	0.08 ± 0.03	0.07 ± 0.03	***	NS	NS
C18:1 9*t*	0.13 ± 0.04	0.10 ± 0.04	0.12 ± 0.04	0.12 ± 0.04	**	NS	NS
C18:1 10*t*	0.12 ± 0.03	0.07 ± 0.03	0.10 ± 0.03	0.09 ± 0.03	**	NS	NS
C18:1 11*t*	1.35 ± 0.07	0.15 ± 0.08	0.74 ± 0.08	0.76 ± 0.08	***	NS	NS
C18:1 12*t*	0.19 ± 0.02	0.08 ± 0.02	0.13 ± 0.02	0.13 ± 0.02	***	NS	NS
C18:1 13,14*t*	0.38 ± 0.05	0.10 ± 0.05	0.24 ± 0.05	0.25 ± 0.05	***	NS	NS
C18:1 15*t*	0.17 ± 0.05	0.09 ± 0.06	0.14 ± 0.06	0.13 ± 0.06	*	NS	NS
C18:1 16*t*	0.17 ± 0.01	0.05 ± 0.01	0.11 ± 0.01	0.11 ± 0.01	***	NS	NS
**∑ FA[10*]**	1962.72 ± 1033.46	2376.72 ± 1049.38	1840.80 ± 1040.75	2498.64 ± 1040.75	NS	NS	NS

[1]Values reported as means ± SEM (standard error to mean). [2]RA; Red Angus, [3]AK; Red Angus x Akaushi, [4]NS; not significant; $p > 0.05$; *$p < 0.05$; **$p < 0.01$ ***$p < 0.001$.

[5]∑ SFA = all saturated FAs (10:0, 12:0, 13:0, 14:0, 15:0, 16:0, 17:0, 18:0, 19:0, 20:0, 22:0)

[6]∑ BCFA = sum of all branched chain FAs (*iso*14:0, *iso*15:0, *anteiso*15:0, *iso*16:0, *iso*17:0, *anteiso*17:0, *iso*18:0)

[7]∑ MUFA = all monounsaturated FAs (14:1, 16:1, 17:1, 18:1, 20:1)

[8]∑ *c*MUFA = 14:1, 17:1, sum of *c*16:1, *c*18:1, and *c*20:1

[9]∑ *t*MUFA = sum of *t*16:1 and *t*18:1

[10*]∑ FA = sum of all FAs in mg/100 g meat

Table S2. Mean concentrations of polyunsaturated fatty acids in beef by diet, breed, and diet × breed interaction (% of total fatty acids)[1]

Fatty acid	*Diet (D)*		*Breed (B)*		*Significance of ANOVA*[4]		
	**GRASS**	**GRAIN**	**RA**[2]	**AK**[3]	**D**	**B**	**D × B**
**∑ PUFA**[5]	8.62 ± 1.69	6.56 ± 1.71	8.06 ± 1.69	7.12 ± 1.69	*	NS	NS
**∑ *n*-3**[6]	3.24 ± 0.35	0.74 ± 0.36	2.11 ± 0.35	1.88 ± 0.35	***	NS	NS
C18:3 *n*-3 (ALA)[7]	1.58 ± 0.10	0.23 ± 0.11	0.93 ± 0.10	0.88 ± 0.10	***	NS	NS
C20:3 *n*-3	0.02 ± 0.00	0.00 ± 0.00	0.01 ± 0.00	0.01 ± 0.00	***	NS	NS
C20:5 n-3 (EPA)[8]	0.59 ± 0.08	0.12 ± 0.09	0.39 ± 0.08	0.32 ± 0.08	***	NS	NS
C22:5 n-3 (DPA)[9]	0.99 ± 0.14	0.36 ± 0.14	0.73 ± 0.14	0.62 ± 0.14	***	NS	NS
C22:6 n-3 (DHA)[10]	0.06 ± 0.03	0.02 ± 0.03	0.04 ± 0.03	0.04 ± 0.03	*	NS	NS
**∑ *n*-6**[11]	5.31 ± 1.32	5.74 ± 1.34	5.88 ± 1.33	5.17 ± 1.33	NS	NS	NS
C18:2 n-6 (LA)[12]	3.62 ± 0.77	3.76 ± 0.78	3.88 ± 0.80	3.51 ± 0.80	NS	NS	NS
C18:3 *n*-6	0.03 ± 0.01	0.02 ± 0.01	0.03 ± 0.01	0.02 ± 0.01	NS	NS	NS
C20:2 *n*-6	0.03 ± 0.01	0.03 ± 0.01	0.03 ± 0.01	0.03 ± 0.01	NS	NS	NS
C20:3 *n*-6	0.26 ± 0.06	0.31 ± 0.06	0.31 ± 0.06	0.26 ± 0.06	NS	NS	NS
C20:4 *n*-6	1.24 ± 0.38	1.33 ± 0.38	1.40 ± 0.38	1.16 ± 0.38	NS	NS	NS
C22:4 *n*-6	0.14 ± 0.11	0.29 ± 0.11	0.23 ± 0.11	0.19 ± 0.10	**	NS	NS
***n*-6:*n*-3 ratio**[13]	1.61 ± 0.39	8.36 ± 0.41	5.04 ± 0.39	4.92 ± 0.39	***	NS	NS
C20:3 *n*-9	0.07 ± 0.02	0.07 ± 0.02	0.07 ± 0.02	0.06 ± 0.02	NS	NS	NS
**∑ CLnA**[14]	0.02 ± 0.00	0.02 ± 0.00	0.02 ± 0.00	0.02 ± 0.00	**	NS	NS
C18:3 9*c*,11*t*,15*t*	0.01 ± 0.00	0.00 ± 0.00	0.00 ± 0.00	0.01 ± 0.00	**	NS	NS
C18:3 9*c*,11*t*,15*c*	0.02 ± 0.00	0.01 ± 0.00	0.02 ± 0.00	0.01 ± 0.00	NS	NS	NS
**∑ AD**[15]	0.92 ± 0.04	0.53 ± 0.05	0.72 ± 0.04	0.72 ± 0.04	***	NS	NS
C18:2 11*t*,15*t*	0.21 ± 0.01	0.08 ± 0.01	0.14 ± 0.01	0.16 ± 0.01	***	NS	NS
C18:2 9*t*,12*t*	0.04 ± 0.02	0.01 ± 0.02	0.03 ± 0.02	0.03 ± 0.02	***	NS	NS
C18:2 9*c*,14*t*/9*c*,13*t*	0.13 ± 0.01	0.07 ± 0.01	0.10 ± 0.01	0.10 ± 0.01	***	NS	NS
C18:2 11*t*,15*c*	0.22 ± 0.01	0.04 ± 0.01	0.13 ± 0.01	0.13 ± 0.01	***	NS	NS
C18:2 9*c*,16*t*	0.23 ± 0.08	0.19 ± 0.08	0.23 ± 0.08	0.20 ± 0.08	NS	NS	NS
C18:2 9*c*,15*c*	0.07 ± 0.01	0.11 ± 0.01	0.09 ± 0.01	0.09 ± 0.01	***	NS	NS
C18:2 12*c*,15*c*	0.02 ± 0.01	0.01 ± 0.01	0.01 ± 0.01	0.01 ± 0.01	**	NS	NS
**∑ CLA**[16]	0.34 ± 0.14	0.16 ± 0.14	0.25 ± 0.14	0.26 ± 0.14	***	NS	NS
C18:2 9*c*,11*t*/9*c*,7*t*	0.24 ± 0.08	0.11 ± 0.08	0.17 ± 0.08	0.18 ± 0.08	***	NS	NS
C18:2 11*t*,13*c*	0.04 ± 0.02	0.02 ± 0.02	0.03 ± 0.02	0.03 ± 0.02	**	NS	NS
C18:2 11*t*,13*t*	0.03 ± 0.02	0.02 ± 0.02	0.03 ± 0.02	0.03 ± 0.02	**	NS	NS
C18:2 *t*,*t*	0.03 ± 0.02	0.02 ± 0.02	0.03 ± 0.02	0.03 ± 0.02	NS	NS	NS

[1]Values reported as means ± SEM (standard error to mean) [2]RA; Red Angus, [3]AK; Red Angus x Akaushi, [4]NS; not significant; $p > 0.05$; *$p < 0.05$; **$p < 0.01$ ***$p < 0.001$.

[5]∑ PUFA = LA + ALA + GLA + Eicosadienoic + Eicosatrienoic + DGLA + Mead + Arachidonic + EPA + DTA + DPA *n*-3 + DHA

[6]∑ *n*-3 = ALA + EPA + DHA + DPA *n*-3 + Eicosatrienoic
[7]ALA; alpha-linolenic acid, [8]EPA; eicosapentaenoic acid, [9]DPA; *n*-3 docosapentaenoic acid , [10]DHA; docosahexaenoic acid

[11]∑ *n*-6 = LA + GLA + Eicosadienoic + DGLA + Arachidonic + DTA

[12]LA; linoleic acid

[13]*n*-6:*n*-3 ratio = ∑ *n*-6/∑ *n*-3

[14]∑ CLnA = sum of conjugated linolenic acid isomers (*c*9, *t*11, *t*15 18:3 + *c*9, *t*11, *c*15 18:3)

[15]∑ Atypical Dienes (AD) = sum of non-conjugated linoleic acid isomers (*t*11, *t*15 18:2 + *t*9, *t*12 18:2 + *c*9, *t*14/*c*9, *t*13 18:2 + *t*11, *c*15 18:2 + *c*9, *t*16 18:2 + *c*9, *c*15 18:2 + *c*12, *c*15 18:2)

[16]∑ CLA = sum of conjugated linoleic acid isomers (*c*9, *t*11/*t*7, *c*9 18:2 + *t*11, *c*13 18:2 + *t*11, *t*13 18:2 + *t*,*t* 18:2)
